# Perceptions of Artificial Nutrition and Hydration at the End of Life Among Healthcare Professionals, Medical Students, and Lay Respondents: A Cross-Sectional Comparative Survey

**DOI:** 10.3390/nu18091404

**Published:** 2026-04-29

**Authors:** Mircea Stoian, Dorin Bica, Horatiu Cioloboc, Nicolae Demenciuc, Andrei Manea, Sergio Rares Bandila, Adina Stoian, Leonard Azamfirei

**Affiliations:** 1 Department of Anesthesiology and Intensive Care Medicine, George Emil Palade University of Medicine, Pharmacy, Science and Technology of Târgu Mureș, 540103 Târgu Mureș, Romania; mircea.stoian@umfst.ro (M.S.); cioloboc.horatiu@yahoo.com (H.C.); leonard.azamfirei@umfst.ro (L.A.); 2 Department of Electrical Engineering and Information Technology, Faculty of Engineering and Information Technology, George Emil Palade University of Medicine, Pharmacy, Science Technology, 540103 Târgu Mureş, Romania; dorin.bica@umfst.ro; 3 Doctoral School of Medicine and Pharmacy, George Emil Palade University of Medicine, Pharmacy, Science, and Technology of Târgu Mureș, Gheorghe Marinescu Street No. 38, 540142 Târgu Mureș, Romania; dr.demenciucnicolae@gmail.com (N.D.); andrei.manea@umfst.ro (A.M.); 4 Department of Radiology, George Emil Palade University of Medicine, Pharmacy, Science, and Technology of Târgu Mureș, Gheorghe Marinescu Street No. 38, 540142 Târgu Mureș, Romania; 5 Orthopedic Surgery and Traumatology Service, Marina Baixa Hospital, Av. Alcalde En Jaume Botella Mayor, 03570 Villajoyosa, Spain; sergiob1976@gmail.com; 6 Department of Pathophysiology, George Emil Palade University of Medicine, Pharmacy, Science and Technology of Târgu Mureș, 540103 Târgu Mureș, Romania

**Keywords:** artificial nutrition and hydration, end-of-life care, palliative care, clinical ethics, decision-making

## Abstract

**Background/Objectives**: Artificial Nutrition and Hydration (ANH) at the end of life remains a clinically and ethically complex intervention. Although international guidelines exist, data regarding the awareness of them and their perceived applicability across different population groups remain limited. This study aimed to evaluate and compare perceptions and attitudes regarding ANH among healthcare professionals, medical students, and lay respondents. **Methods**: A cross-sectional, questionnaire-based comparative survey was conducted between July 2025 and March 2026, including 470 respondents (338 healthcare professionals, 46 medical students, and 86 lay respondents). The survey assessed perceptions of ANH, factors influencing decision-making, and familiarity with clinical guidelines and legislation. **Results**: General perceptions regarding ANH were broadly similar across groups. Significant differences were observed for the importance assigned to estimated life expectancy (*p* < 0.001) and family opinion (*p* = 0.017). Associations were identified between study group and opinions on clinical guidelines (χ^2^(6) = 16.366, *p* = 0.012) and legislation (χ^2^(6) = 14.712, *p* = 0.023), with lack of knowledge more frequent among lay respondents and students. Within healthcare professionals, physicians and nurses showed significantly different responses regarding guidelines (*p* < 0.001). **Conclusions**: In this cross-sectional survey, perceptions of ANH at the end of life were largely shared, but differed in relation to prognostic factors, family involvement, and awareness of guidelines and legislation, suggesting the presence of relevant knowledge gaps in end-of-life decision-making.

## 1. Introduction

Artificial Nutrition and Hydration (ANH) represent essential components of supportive medical care in patients who are unable to maintain adequate oral intake due to acute or chronic illness. Nutritional support, including enteral and parenteral nutrition, is widely used across multiple clinical specialties, particularly in intensive care medicine, oncology, geriatrics, and palliative care.

When appropriately indicated, nutritional therapy contributes to the prevention and treatment of disease-related malnutrition, the improvement of functional status, and reduction in complications associated with inadequate nutritional intake [1,2,3,4].

In contrast, the role of Artificial Nutrition and Hydration becomes more complex when patients approach the end of life. In this context, decisions regarding the initiation, continuation, withholding, or withdrawal of ANH are often influenced not only by clinical considerations but also by ethical principles, cultural values, and societal expectations [5,6]. These decisions frequently arise in situations characterized by medical uncertainty, limited life expectancy, and evolving goals of care.

International professional societies emphasize that ANH should be considered a medical intervention rather than basic care. As such, it requires a clear clinical indication, defined therapeutic goals, and ongoing evaluation of proportionality between expected benefits and potential burdens. According to current guidelines, decisions regarding ANH should respect the principles of biomedical ethics, including patient autonomy, beneficence, non-maleficence, and justice [2,6,7,8].

The clinical effectiveness of artificial nutrition in advanced illness remains the subject of considerable debate. Evidence from studies involving patients with advanced dementia suggests that enteral feeding does not consistently improve survival, prevent aspiration pneumonia, or enhance quality of life [9,10]. Similarly, observational research has demonstrated that feeding tube placement in patients with severe cognitive impairment may be associated with complications without providing substantial clinical benefit [10]. These findings have raised important questions regarding the appropriateness of invasive nutritional interventions in patients with limited life expectancy.

Beyond clinical considerations, decisions regarding Artificial Nutrition and Hydration at the end of life are strongly influenced by ethical, cultural, and emotional factors. Feeding is widely perceived as a fundamental expression of care and compassion, and limiting or withdrawing nutritional support may therefore be interpreted by families or members of the public as abandonment of the patient [11,12,13].

These perceptions may create tension between the medical evaluation of treatment proportionality and the societal perception of feeding as a basic human obligation. Previous studies have shown that differences in the understanding of life-sustaining treatments, prognostic uncertainty, and emotional distress can complicate communication and shared decision-making in end-of-life care [14,15,16].

Large international studies evaluating end-of-life practices have also demonstrated significant variability in attitudes toward life-sustaining treatments, including ANH. For example, research conducted in European intensive care units has revealed considerable differences in clinical approaches to treatment limitation and withdrawal of life-sustaining therapies [17,18,19,20]. Such variability may reflect differences in professional training, ethical perspectives, cultural contexts, and healthcare system characteristics. These considerations are particularly relevant in Eastern European settings, including Romania, where cultural, religious, and legal factors may further shape attitudes toward end-of-life decision-making.

However, relatively few studies have simultaneously examined perceptions of Artificial Nutrition and Hydration among healthcare professionals, medical students, and members of the general population. Understanding how these groups perceive nutritional interventions at the end of life is particularly important because differences in medical knowledge, ethical reasoning, and personal beliefs may influence clinical communication, shared decision-making, and acceptance of treatment limitation [15,21].

Furthermore, familiarity with ethical guidelines and legal frameworks governing decisions related to ANH remains insufficiently explored. Assessing perceptions across groups with different levels of medical training may help identify knowledge gaps and educational needs related to end-of-life nutritional decision-making [2,22].

Therefore, the aim of the present study was to evaluate and compare perceptions, attitudes, and self-reported preparedness regarding Artificial Nutrition and Hydration at the end of life among healthcare professionals, medical students, and lay respondents.

## 2. Materials and Methods

### 2.1. Study Design and Setting

This study was designed as a cross-sectional, questionnaire-based comparative survey aimed at evaluating perceptions and attitudes regarding Artificial Nutrition and Hydration (ANH) at the end of life. Data collection was conducted between July 2025 and March 2026 through an anonymous online questionnaire distributed via professional medical communication groups, academic student channels, and publicly accessible online platforms. The study was specifically designed to compare perceptions among respondents with different levels of medical education and clinical exposure.

### 2.2. Participants and Eligibility Criteria

Participants were recruited from three predefined categories: healthcare professionals, including physicians, nurses, and other medical staff involved in patient care; undergraduate medical students predominantly enrolled in the general medicine program, with a small proportion from other health-related fields such as nutrition; and lay respondents from the general population without formal medical training or professional roles in healthcare.

Eligibility criteria included age of at least 18 years, provision of electronic informed consent, and completion of the full questionnaire. Incomplete questionnaires and duplicate submissions were excluded from the final analysis. Participation was voluntary, anonymous, and no financial or material incentives were provided.

Because the survey was distributed through open online channels, the exact number of eligible individuals reached could not be determined and no formal response rate could be calculated. This recruitment approach may increase the risk of selection bias and limits the assessment of sample representativeness.

### 2.3. Questionnaire Development and Structure

The questionnaire was developed by a multidisciplinary team including specialists in anesthesiology and intensive care, clinical nutrition, and pathophysiology, based on the current literature, relevant ethical–legal frameworks, and ESPEN/ASPEN guidelines. Its content and structure were reviewed within the research team, and the statistical design was revised by the study statistician prior to dissemination.

The survey included items on demographic and professional characteristics, previous exposure to end-of-life care, perceived benefits and burdens of ANH, ethical attitudes toward withholding or withdrawal, factors influencing decision-making, and familiarity with clinical guidelines and legislation.

Most attitudinal items were assessed using 5-point Likert scales ranging from strongly disagree to strongly agree or from not important to very important, depending on the item format. In addition, the questionnaire included multiple-choice categorical questions exploring personal preferences and opinions regarding decision-making responsibilities. For the purpose of this study, ANH was defined as a medical intervention including enteral and parenteral nutrition, as well as medically assisted hydration.

ANH was approached as a combined construct because this terminology is commonly used in ethical guidance and in the end-of-life literature, where Artificial Nutrition and Hydration are often discussed within a shared decision-making framework.

The full English version of the questionnaire used in this study is provided in the Supplementary File S1—Questionnaire.

### 2.4. Terminology

For the purposes of this study, Artificial Nutrition and Hydration (ANH) were defined as medical interventions used to provide nutrients and fluids when adequate oral intake cannot be maintained. Artificial nutrition included both enteral nutrition delivered through feeding tubes, such as nasogastric tubes or gastrostomy, and parenteral nutrition administered intravenously. Artificial hydration referred to the administration of fluids through intravenous or subcutaneous routes.

In contrast, oral feeding or comfort feeding was defined as the provision of food and fluids by mouth as tolerated by the patient and was considered a component of basic care rather than a medical intervention. This conceptual distinction was considered important when formulating the questionnaire in order to avoid potential ambiguity between supportive care and medical treatment.

### 2.5. Ethical Considerations

The study protocol was reviewed and approved by the Ethics Committee of the University of Medicine, Pharmacy, Science and Technology “George Emil Palade” of Târgu Mureș (approval no. 3883/20.10.2025). The survey instrument was reviewed as part of the approved study protocol.

In addition, the study received approval from the Ethics Committee of the Mureș County Clinical Hospital, decision no. 9916/22.07.2025.

Before accessing the questionnaire, all participants were provided with information regarding the purpose of the study, the voluntary nature of participation, data confidentiality, and the anonymous processing of responses.

Informed consent was implied through voluntary agreement to participate and completion of the questionnaire. All data were collected anonymously in accordance with GDPR regulations and institutional ethical standards.

### 2.6. Study Variables and Sampling Strategy

The main study variables included respondent group, age, sex, religious affiliation, previous exposure to end-of-life care, self-reported ethical–legal knowledge, and attitudes regarding the initiation, continuation, and discontinuation of ANH in end-of-life clinical scenarios.

Particular attention was given to responses related to the perceived justification of initiating ANH in emergency situations without explicit consent, continuing ANH in unconscious patients at the request of family members, and discontinuing ANH in unconscious or emergency clinical settings.

Additional variables included perceptions regarding the clinical usefulness of ANH, the importance attributed to prognostic and ethical decision-making factors, and familiarity with professional guidelines and legal frameworks.

Participants were recruited using a convenience sampling strategy. No formal a priori sample size calculation was performed. Given the exploratory design and open online distribution of the survey, the final sample size was determined by the number of complete responses obtained during the study period.

Only fully completed questionnaires were included in the final analysis; incomplete responses were excluded, and no missing data imputation was performed.

Given the unequal distribution of participants across groups, particularly the smaller medical student subgroup, the study may have had limited power to detect small between-group differences.

### 2.7. Statistical Analysis

Statistical analyses were performed using IBM SPSS Statistics for Windows, version 31.0 (IBM Corp., Armonk, NY, USA).

Categorical variables were summarized as absolute frequencies and percentages. Continuous and ordinal variables were assessed for normality using the Shapiro–Wilk test and are presented as median and interquartile range (IQR) or mean ± standard deviation (SD), as appropriate.

Comparisons between the three study groups were performed using the chi-square test for categorical variables, the Kruskal–Wallis test for ordinal Likert-scale variables and non-normally distributed continuous variables, and one-way ANOVA for normally distributed continuous variables. When statistically significant differences were identified, post hoc pairwise comparisons with Bonferroni’s correction were performed.

A *p*-value of less than 0.05 was considered statistically significant. *p*-values were reported according to the software output, with very small values displayed and reported as *p* < 0.001.

## 3. Results

### 3.1. Participant Characteristics

A total of 470 respondents completed the questionnaire and were included in the final analysis, comprising 338 healthcare professionals, 46 medical students, and 86 lay respondents. Healthcare professionals represented the predominant respondent group, accounting for nearly three quarters of the total study population.

A statistically significant difference in age distribution was observed across the three groups (*p* < 0.001). Healthcare professionals had a median age of 41 years (IQR 33–50), lay respondents 50.5 years (IQR 43–68), and medical students 23 years (IQR 22–25). No statistically significant difference was found regarding sex distribution between groups (*p* = 0.311). Previous exposure to end-of-life care differed significantly across groups (*p* < 0.001), being reported by 82.5% of healthcare professionals, 19.56% of medical students, and 74.4% of lay respondents. This exposure most likely reflects personal experiences, such as caring for or accompanying close relatives during end-of-life situations.

Religious affiliation did not differ significantly among groups (*p* = 0.122).

The demographic and professional characteristics of the participants are presented in Table 1.

### 3.2. Perceptions Regarding Artificial Nutrition and Hydration at the End of Life

Responses to the Likert-scale items assessing perceptions regarding the clinical role and ethical acceptability of Artificial Nutrition and Hydration at the end of life are presented in Table 2.

No statistically significant between-group differences were observed for the following items: artificial nutrition prolongs life (*p* = 0.152), artificial nutrition improves quality of life at the end of life (*p* = 0.771), artificial nutrition prevents complications such as bedsores or infections (*p* = 0.350), artificial nutrition is a necessary care gesture regardless of the patient’s condition (*p* = 0.941), continuing artificial nutrition in an unconscious patient is justified if the family requests (*p* = 0.856), stopping artificial nutrition is equivalent to euthanasia (*p* = 0.776), initiating artificial nutrition without consent is justifiable in emergencies (*p* = 0.756), and stopping artificial nutrition without consent is justifiable in emergencies (*p* = 0.383).

A statistically significant difference was observed for the item “stopping artificial nutrition in an unconscious patient is justified if the family requests” (*p* = 0.018, eta-squared= 0.013, indicating a small effect). Median responses for this item were 4 (IQR 2–4) among lay respondents, 3.5 (IQR 2–4) among medical students, and 3 (IQR 2–4) among healthcare professionals.

The distribution of responses is presented in Table 2.

Additional correlation analyses showed very weak negative correlations between age and the perception that artificial nutrition improves quality of life at the end of life (r = −0.122, *p* = 0.025), as well as between years of professional experience and the same item (r = −0.119, *p* = 0.029).

Very weak positive correlations were identified between age and the opinion that artificial nutrition is useless in terminal patients (r = 0.123, *p* = 0.024), and between years of professional experience and this item (r = 0.113, *p* = 0.038).

### 3.3. Factors Influencing Decision-Making Regarding Artificial Nutrition and Hydration

Participants were asked to rate the importance of several clinical and ethical factors involved in decision-making regarding Artificial Nutrition and Hydration at the end of life. The results are presented in Table 3.

No statistically significant between-group differences were observed for nutritional status (*p* = 0.751), patient’s expressed wishes (*p* = 0.294), quality of life (*p* = 0.351), comorbidities (*p* = 0.290), risk of pulmonary aspiration (*p* = 0.553), and type of nutrition (enteral/parenteral) (*p* = 0.130).

Statistically significant between-group differences were identified for estimated life expectancy (*p* < 0.001, eta-sqared= 0.026) and family opinion (*p* = 0.017, eta-sqared = 0.013), with eta-squared values indicating a small effect.

For estimated life expectancy, the median response was 4 (IQR 3–5) among lay respondents, 5 (IQR 4–5) among medical students, and 4 (IQR 3–5) among healthcare professionals, with statistically significant differences between healthcare professionals and students and between students and lay responders.

For family opinion, the median response was 3 (IQR 2–4) among lay respondents, 4 (IQR 3–4) among medical students, and 4 (IQR 3–4) among healthcare professionals, with statistically significant differences between healthcare professionals and lay responders and between students and lay responders.

Post hoc pairwise comparisons for variables with statistically significant overall differences were performed using Bonferroni’s correction and are summarized in the table notes.

A supplementary analysis comparing respondents with and without prior experience in artificial nutrition showed a statistically significant difference regarding the importance assigned to estimated life expectancy in decision-making (Mann–Whitney U test, *p* = 0.036).

Respondents without prior experience in artificial nutrition had higher scores for this item compared with those with prior experience.

### 3.4. Opinions on Clinical Guidelines and Legislation Regarding ANH

A statistically significant association was observed between study group and opinion regarding clinical guidelines related to Artificial Nutrition and Hydration at the end of life (χ^2^(6) = 16.366, *p* = 0.012). A Cramer’s V of 0.13 (95% CI 0.030–0.178) suggests a small effect size. The category “lack of knowledge” was most frequently reported among lay respondents and medical students, whereas healthcare professionals more frequently selected responses classified as “moderately positive (useful but limited)”.

The distribution of responses is presented in Table 4.

A statistically significant association was also identified between study group and opinion regarding the legal framework governing end-of-life nutritional decisions (χ^2^(6) = 14.712, *p* = 0.023), while a Cramer’s V of 0.14 (95% CI 0.01–0.17) suggests a small effect size. For legislation-related responses, the category “lack of knowledge” was most frequent among lay respondents and medical students, whereas healthcare professionals more frequently selected responses classified as “unclear/requires improvement”.

The distribution of responses regarding legislation is presented in Table 5.

### 3.5. Secondary Subgroup Analysis Among Healthcare Professionals

A secondary subgroup analysis was performed within the healthcare professional cohort and was restricted to physicians (n = 275) and nurses (n = 63), as the two main professional categories directly involved in ANH-related clinical decision-making.

A statistically significant association was observed between professional status and opinion regarding clinical guidelines (χ^2^(3) = 17.04, *p* < 0.001), while a Cramer’s V = 0.23 (95% CI 0.10–0.32) suggests a small-to-moderate association between the variables.

Physicians more frequently selected responses classified as “moderately positive”, whereas nurses more frequently selected responses classified as “negative (opposition)”.

The frequency of responses classified as “lack of knowledge” was comparable between the two professional categories.

The distribution of responses is presented in Table 6.

## 4. Discussion

The present cross-sectional comparative survey explored perceptions of Artificial Nutrition and Hydration (ANH) at the end of life among healthcare professionals, medical students, and lay respondents from a regional cohort in central Transylvania.

Overall, the findings suggest that perceptions regarding the general role of ANH were broadly comparable across groups. However, some statistically significant differences emerged in relation to the factors influencing decision-making, the awareness of clinical guidelines and legal frameworks, and professional subgroup attitudes [6,23].

This overall pattern should also be considered in the context of the study composition, given that healthcare professionals represented the predominant respondent group while the student subgroup was comparatively smaller, which may have influenced between-group comparisons. However, these differences should be interpreted with caution, as several were associated with modest effect sizes and may not reflect clinically meaningful distinctions. These findings should be interpreted within the exploratory framework of the study.

From an ethical perspective, the results should be interpreted within the current international framework, which consistently defines ANH as a medical intervention requiring indication, proportionality, and alignment with patient-centered goals of care [2,24,25]. The tension between evidence-based proportionality and the moral symbolism of feeding has been repeatedly emphasized in recent reviews and ethical position papers [25,26].

### 4.1. General Perceptions Regarding Artificial Nutrition and Hydration at the End of Life

In the present study, perceptions regarding the general clinical role of Artificial Nutrition and Hydration (ANH) were broadly similar across healthcare professionals, medical students, and lay respondents, with no statistically significant between-group differences for most of the general attitudinal items. This relative convergence may suggest that ANH continues to be perceived not only as a clinical intervention but also as a symbolically charged aspect of end-of-life care.

This observation is consistent with the position expressed by the ESPEN expert panel, who explicitly state that ANH should be regarded as a medical treatment, the use of which must depend on indication, proportionality, expected benefit, and patient-centered goals [6]. Importantly, the authors emphasize that one of the major challenges in clinical practice is the frequent confusion between ANH as a medical intervention and feeding as a basic human gesture of care and compassion [6].

In our cohort, age and years of professional experience showed only very weak associations with ANH-related attitudes, whereas the recent literature suggests that clinical role and prior palliative care exposure may be more influential determinants of end-of-life decision-making [25,27].

A similar perspective is advanced in the ASPEN position, which states that artificially administered nutrition and hydration should be evaluated according to the same ethical standards as any other medical treatment, particularly with regard to autonomy, beneficence, and proportionality [26]. The position paper further underlines that conflicts may arise because patients and families often perceive nutrition and hydration primarily through an emotional and relational framework, while clinicians are expected to apply evidence-based medical reasoning [26].

The importance of patient wishes and family involvement is also reflected in other severe neurological conditions, where long-term life-sustaining interventions may be continued in accordance with clearly expressed patient goals. For instance, Stoian et al. described prolonged home mechanical ventilation in a patient with Amyotrophic lateral sclerosis, highlighting the central role of patient determination, family support, and shared decision-making in complex care pathways [28].

### 4.2. Family Involvement and Prognosis as Key Factors in ANH Decision-Making

Among all decision-making factors evaluated, estimated life expectancy and family opinion emerged as the key variables differentiating responses across the three study groups.

Medical students assigned greater importance to estimated life expectancy, whereas healthcare professionals and students attributed more weight to family opinion compared with lay respondents.

The important role attributed to family opinion is consistent with the current literature and international guidance. Both ESPEN and ASPEN emphasize that, particularly in patients with impaired decision-making capacity, family involvement is an essential component of the decision-making process, while maintaining the primacy of patient autonomy and previously expressed wishes [6,26].

Similar findings have been reported in recent studies, where family expectations, concerns about comfort, and fear of abandonment significantly influenced ANH-related decisions at the end of life [19,25].

This interpretation is supported by the supplementary analysis, which indicated that respondents without prior experience in artificial nutrition assigned greater importance to estimated life expectancy. These findings are consistent with previous studies suggesting that direct clinical exposure to end-of-life care may influence the relative importance assigned to prognostic factors and treatment proportionality [11,25,27,29].

Together, these findings suggest that both clinical prognosis and relational decision-making play an important role in attitudes toward ANH at the end of life.

### 4.3. Clinical Guidelines, Legal Uncertainty, and Applicability in Practice

A major finding of the present study was the limited familiarity with clinical guidelines and the frequent perception of the legal framework as unclear or insufficiently defined, including among healthcare professionals.

This aligns with the recent literature indicating that decision-making regarding Artificial Nutrition and Hydration (ANH) at the end of life is strongly influenced by variability in ethical norms, legislation, and local institutional practices.

Although international societies such as ESPEN and ASPEN provide clear ethical frameworks, their practical implementation differs substantially across countries and healthcare systems [6,26].

The recent literature highlights that ANH decisions are frequently affected by the absence of harmonized legal standards. For example, a recent qualitative hospice study showed that decision-making trajectories are shaped not only by medical factors but also by social, legal, and cultural constraints. Family caregivers frequently experience uncertainty and moral distress [30,31,32,33]. Similarly, the literature consistently indicates that decision-making regarding ANH at the end of life is a dynamic and multifactorial process, often complicated by uncertainty related to patient autonomy, professional duties, family expectations, and the legal framework governing treatment limitation [34,35,36,37].

In some jurisdictions, such as the United Kingdom, clinically assisted nutrition and hydration (CANH) is recognized as a medical treatment and may be withheld or withdrawn within an established legal and professional framework, as detailed in the GMC and BMA/RCP guidance for England and Wales [38,39]. Importantly, UK guidance clearly distinguishes withholding or withdrawing CANH from euthanasia and emphasizes best-interest decision-making when patients lack capacity.

Comparable legal approaches have also been reported in other European countries, including France and the Netherlands, where ANH decisions are addressed within broader legal and ethical frameworks related to treatment limitation and patient autonomy [40,41,42].

By contrast, in several healthcare systems, including parts of Eastern Europe, the legal framework specifically addressing ANH at the end of life remains less explicit or insufficiently detailed. In such contexts, clinical decision-making is often supported by international professional guidelines, institutional ethics committees, and multidisciplinary consensus rather than by specific statutory regulation [6,26,43].

Taken together, these findings highlight the need for clearer institutional guidance and structured ethical–legal education regarding ANH-related decision-making at the end of life.

### 4.4. Additional Subgroup Analysis Among Healthcare Professionals

The secondary subgroup analysis restricted to physicians and nurses showed a statistically significant association between professional status and opinion regarding clinical guidelines related to ANH.

More specifically, physicians more frequently expressed a moderately positive attitude, considering guidelines useful but with certain limitations, whereas nurses more often selected responses corresponding to opposition or lower confidence in guideline applicability.

This finding may reflect differences in professional roles, the level of responsibility in end-of-life decision-making, and the degree of exposure to ethical and legal guidance. Previous studies have similarly shown that physicians and nurses may differ in their perceptions of ANH, particularly with regard to treatment goals, proportionality, and end-of-life limitation of care [25,44,45].

These differences may reflect role-specific perspectives in end-of-life care. Recent evidence suggests that physicians and nurses may differ not only in the perceived appropriateness of ANH, but also in the relative weight assigned to prognosis, patient comfort, and family expectations. In particular, nurses tend to report greater concern regarding comfort-related aspects and day-to-day care burden, whereas physicians more frequently frame decisions within prognosis-based therapeutic proportionality and treatment goals [25,46,47].

Moreover, palliative care experience has been shown to possibly influence healthcare professionals’ beliefs and decision-making regarding ANH. It may improve adherence to evidence-based recommendations and reduce misconceptions related to survival benefit or quality-of-life improvement [2,25,48].

### 4.5. Strengths, Limitations, and Future Directions

The present study has several strengths. To our knowledge, this is among the few comparative surveys exploring perceptions of ANH at the end of life across healthcare professionals, medical students, and lay respondents within the same regional population. This comparative design allowed the identification of both convergent and divergent attitudes across groups directly involved in or affected by end-of-life decision-making.

At the same time, several limitations should be acknowledged.

The cross-sectional design captures perceptions at a single time point and does not allow causal inferences or the assessment of how attitudes may evolve with increasing clinical experience, education, or exposure to palliative care.

No formal a priori sample size calculation was performed. As this study was exploratory and based on convenience sampling, the final sample size was determined pragmatically by the number of complete responses collected during the recruitment period. This approach may have limited the statistical power for some between-group comparisons, particularly in the context of unequal group sizes.

The imbalance between groups, particularly the smaller medical student subgroup, means that the study was more likely to detect moderate-to-large effects rather than small between-group differences.

This study was conducted on a regional cohort from central Transylvania, which may limit the generalizability of the findings to other Romanian regions or to healthcare systems with different cultural, legal, and institutional contexts.

Because the survey was distributed through open online channels, the total number of eligible individuals reached could not be determined and no formal response rate could be calculated. This recruitment approach increases the risk of selection bias and limits the assessment of sample representativeness.

Responses were based on self-reported perceptions and hypothetical decision-making scenarios, which may not fully reflect real-life clinical behavior in emotionally charged end-of-life situations. In addition, the questionnaire did not undergo formal validation or reliability testing, which may limit the robustness and reproducibility of the findings.

This study did not include a more detailed stratification of medical students according to their stage of training or specific field of study, which may have influenced the interpretation of this subgroup.

Although religious affiliation was recorded, this study did not include a deeper evaluation of religiosity, spirituality, or cultural beliefs, factors that may substantially influence attitudes toward Artificial Nutrition and Hydration (ANH) and end-of-life care, as emphasized in the recent palliative care literature [49,50].

Another limitation is that the questionnaire addressed Artificial Nutrition and Hydration largely as a combined construct. Although this reflects commonly used terminology in the literature and ethical guidance, the two interventions may be perceived differently in clinical, emotional, and moral terms.

Finally, the study population was predominantly composed of healthcare professionals, which may have influenced the overall distribution of attitudes and may limit the generalizability of the findings to the general population. In addition, although lay respondents were defined as individuals without formal medical training, this study did not include a more detailed characterization of their personal caregiving experience or other relevant backgrounds.

Future studies should expand this work through multicenter national surveys, qualitative interviews, and interdisciplinary comparisons, with particular attention to cultural values, family expectations, professional training, and legal awareness in end-of-life ANH decision-making. Further research should explore the role of structured educational interventions in undergraduate and postgraduate medical curricula aimed at improving ethical and clinical decision-making in this context.

### 4.6. Practical Implications

Despite these limitations, the present findings may have several practical implications for end-of-life care and professional education.

As this study was based on a questionnaire exploring perceptions and attitudes, the results should be interpreted primarily as reflecting current views, knowledge gaps, and decision-making tendencies, rather than actual clinical behavior.

Nevertheless, the limited familiarity with guidelines and the frequent perception of legal uncertainty identified in our cohort suggest the need for better educational support, particularly in the areas of ethics, legislation, and communication related to Artificial Nutrition and Hydration (ANH) at the end of life.

In addition, the significant importance attributed to family opinion may support the development of structured communication pathways and shared decision-making frameworks, especially in multidisciplinary settings [2,6,26,51].

## 5. Conclusions

This cross-sectional comparative survey suggests that perceptions regarding Artificial Nutrition and Hydration (ANH) at the end of life are shaped by both shared attitudes and group-specific differences.

Although general perceptions were broadly similar across healthcare professionals, medical students, and lay respondents, family opinion and estimated life expectancy emerged as the most relevant differentiating factors in decision-making.

This study also indicates limited familiarity with clinical guidelines and a frequent perception of legal uncertainty, including among healthcare professionals, suggesting potential educational and institutional gaps in end-of-life ANH-related decision-making.

Moreover, the differences observed between physicians and nurses in guideline-related responses underline the importance of professional role, training, and multidisciplinary communication in ethically complex care pathways.

Overall, these findings suggest that decision-making regarding ANH at the end of life remains a complex multidimensional process shaped by clinical, ethical, familial, and professional factors, within the context of a regional sample and the limitations inherent to the study design.

## Figures and Tables

**Table 1:** Demographic and professional characteristics of participants.

Variable	Healthcare Professionals (n = 338)	Medical Students (n = 46)	Lay Respondents (n = 86)	*p* (Post hoc Group, *p*)
Age, median (IQR)	41 (33–50)	23 (22–25)	50.5 (43–68)	<0.001 (1–2, <0.001; 1–3, <0.001; 2–3, <0.001)
Sex (female, %)	203 (60)	33 (71.73)	53 (61.62)	0.311
Previous exposure to end-of-life care (%)	279 (82.5)	9 (19.56)	64 (74.4)	<0.001 (1–2, <0.001); 1–3, 0.087; 2–3, <0.001)
Religious affiliation (%)				
Orthodox	226 (66.9)	33 (71.73)	56 (65.1)	0.122
Other Christian religions	100 (29.6)	8 (17.39)	26 (30.2)	
Atheist	12 (3.6)	5 (10.86)	4 (4.7)	
Self-reported ethical–legal knowledge (%)				
Yes	80 (23.7)	9 (19.56)	22 (25.6)	0.132
No	115 (34)	18 (39.13)	40 (46.5)	
Not sure	143 (42.3)	19 (41.3)	24 (27.9)	

Note: Continuous variables are presented as median (IQR). Categorical variables are presented as n (%). *p*-values were calculated using the Kruskal–Wallis test for continuous variables and chi-square test for categorical variables. For variables with statistically significant overall differences, post hoc pairwise comparisons with Bonferroni’s correction were performed. Group labels: 1 = healthcare professionals, 2 = medical students, 3 = lay respondents.

**Table 2:** Perceived clinical role of artificial nutrition and hydration.

Item	Overall Median (IQR)	Healthcare Professionals (n = 338)	Medical Students (n = 46)	Lay Respondents (n = 86)	*p* (Post hoc Group, *p*)
Artificial nutrition prolongs life	4 (3–4)	4 (3–4)	4 (3–4)	4 (3–5)	0.152
Artificial nutrition improves quality of life at the end of life	4 (2–4)	4 (2–4)	3 (2–4)	3 (2–4)	0.771
Artificial nutrition prevents complications such as bedsores or infections	3 (2–4)	3 (2–4)	3 (2–4)	3 (2–4)	0.35
Artificial nutrition is a necessary care gesture, regardless of the patient’s condition	4 (2–4)	4 (2–4)	3 (2–4)	4 (2–4)	0.941
Continuing artificial nutrition in an unconscious patient is justified if the family requests	3 (2–4)	3 (2–4)	3 (2–4)	3 (2–4)	0.856
Stopping artificial nutrition in an unconscious patient is justified if the family requests	3 (2–4)	3 (2–4)	3.5 (2–4)	4 (2–4)	0.018 (1–2, 0.096; 1–3, 0.094; 2–3, 1)
Stopping artificial nutrition is equivalent to euthanasia	2 (1–3)	2 (1–3)	2 (1–3)	2 (1–3)	0.776
Initiating artificial nutrition without consent is justifiable in emergencies	4 (3–5)	4 (3–5)	4 (3–5)	4 (3–5)	0.756
Stopping artificial nutrition without consent is justifiable in emergencies	3.5 (3–4)	3.5 (3–4)	3 (2–4)	4 (2–4)	0.383

Note: Data are presented as median (IQR). Between-group comparisons were performed using the Kruskal–Wallis test. For items with statistically significant overall differences, post hoc pairwise comparisons with Bonferroni’s correction were applied. Group labels: 1 = healthcare professionals, 2 = medical students, 3 = lay respondents. Statistical significance was defined as *p* < 0.05.

**Table 3:** Factors associated with decision-making regarding artificial nutrition and hydration.

Factor	Overall Median (IQR)	Healthcare Professionals (n = 338)	Medical Students (n = 46)	Lay Respondents (n = 86)	*p* (Post hoc Group, *p*)
Nutritional status	4 (4–5)	4 (4–5)	4 (4–5)	5 (4–5)	0.751
Estimated life expectancy	4 (3–5)	4 (3–5)	5 (4–5)	4 (3–5)	<0.001 (1–2, 0.001; 1–3, 1; 2–3, 0.001)
Patient’s expressed wishes	4 (4–5)	4 (4–5)	5 (4–5)	4 (4–5)	0.294
Family opinion	4 (2–4)	4 (3–4)	4 (3–4)	3 (2–4)	0.017 (1–2, 0.786; 1–3, 0.047; 2–3, 0.031)
Quality of life	5 (4–5)	5 (4–5)	5 (4–5)	5 (4–5)	0.351
Comorbidities	4 (4–5)	4 (4–5)	4 (4–5)	4 (4–5)	0.290
Risk of pulmonary aspiration	4 (4–5)	4 (4–5)	5 (4–5)	5 (4–5)	0.553
Type of nutrition (enteral/parenteral)	4 (4–5)	4 (4–5)	4 (4–5)	4 (3–5)	0.130

Note: Data are presented as median (IQR). Between-group comparisons were performed using the Kruskal–Wallis test. For variables with statistically significant overall differences, post hoc pairwise comparisons with Bonferroni’s correction were performed. Group labels: 1 = healthcare professionals, 2 = medical students, 3 = lay respondents. Statistical significance was defined as *p* < 0.05.

**Table 4:** Opinions on clinical guidelines regarding artificial nutrition and hydration.

Opinion Category	Healthcare Professionals (n = 338)	Medical Students (n = 46)	Lay Respondents (n = 86)	Total
Lack of knowledge	142 (42)	28 (60.9)	54 (62.8)	224
Negative (opposition)	28 (8.3)	3 (6.5)	4 (4.7)	35
Positive (guideline acceptance)	37 (10.9)	5 (10.9)	7 (8.1)	49
Moderately positive (useful but limited)	131 (38.8)	10 (21.7)	21 (24.4)	162

Note: Data are presented as n. The association between study group and opinion on clinical guidelines was assessed using the chi-square test (χ^2^(6) = 16.366, *p* = 0.012; Cramer’s V = 0.132 (95% CI 0.030–0.178)).

**Table 5:** Opinion on legislation regarding ANH.

Opinion Category	Healthcare Professionals (n = 338)	Medical Students (n = 46)	Lay Respondents (n = 86)	Total
Unclear/requires improvement	128 (37.9)	12 (26.1)	18 (20.9)	158
Lack of knowledge	142 (42.3)	24 (52.2)	54 (62.8)	221
Negative	56 (16.6)	8 (17.4)	10 (11.6)	74
Positive	11 (3.3)	2 (4.3)	4 (4.7)	17

Note: Data are presented as n. The association between study group and opinion on legislation was assessed using the chi-square test (χ^2^(6) = 14.712, *p* = 0.023); Cramer’s V = 0.14 (95% CI 0.01–0.17).

**Table 6:** Opinion on clinical guidelines according to professional status among physicians and nurses included in the subgroup analysis (n = 338).

Professional status	Nurse (n = 63)	Physician (n = 275)	Total
Lack of knowledge	26 (41.3)	116 (42.2)	142
Negative (opposition)	13 (20.6)	15 (5.5)	28
Positive	7 (11.1)	30 (10.9)	37
Moderately positive	17 (27)	114 (41.5)	131

Note: Data are presented as n. Response categories were grouped as follows: “lack of knowledge” = respondents reporting no familiarity with clinical guidelines; “negative (opposition)” = respondents expressing disagreement or opposition to the usefulness or applicability of guidelines; “positive” = respondents reporting clear acceptance of guidelines; “moderately positive” = respondents considering guidelines useful but with certain limitations. The association between professional status and opinion on clinical guidelines was assessed using the chi-square test (χ^2^(3) = 17.04, *p* < 0.001, Cramer’s V = 0.23 (95% CI 0.10–0.32)).

## Data Availability

Data are available based on request from the corresponding author. The data are not publicly available due to privacy and ethical restrictions.

## Supplementary Materials

The following supporting information can be downloaded at: https://www.mdpi.com/article/10.3390/nu18091404/s1. Supplementary File S1, English version of the study questionnaire.

## Abbreviations

ANH	Artificial Nutrition and Hydration
ICU	Intensive care unit
IQR	Interquartile range
ESPEN	European Society for Clinical Nutrition and Metabolism
ASPEN	American Society for Parenteral and Enteral Nutrition
CANH	Clinically assisted nutrition and hydration
SD	Standard deviation
