# Supplementary material for: Perceptions of Artificial Nutrition and Hydration at the End of Life Among Healthcare Professionals, Medical Students, and Lay Respondents: A Cross-Sectional Comparative Survey
Source: Nutrients. 2026 Apr 29;18(9):1404. doi: 10.3390/nu18091404 (PMC13164728; doi:10.3390/nu18091404)
Supplement: Supplementary file 1 [file nutrients-18-01404-s001.zip › nutrients-4283146-supplementary.pdf]

Supplementary File – Questionnaire (English Version)

SECTION 1. DEMOGRAPHIC DATA

Age:
[Free text]

Sex:

☐ Male

☐ Female

Religion:

☐ Orthodox

☐ Roman Catholic

☐ Greek Catholic

☐ Reformed

☐ Baptist

☐ Pentecostal

☐ Islam

☐ Jewish

☐ Atheist/Agnostic

☐ Prefer not to answer

☐ Other: __________

SECTION 2. PROFESSIONAL ROLE AND EXPERIENCE

What is your role in relation to the care of terminally ill patients?

☐ Physician

☐ Nurse

☐ Support staff (e.g., nursing assistant)

☐ Patient

☐ Relative / legal guardian

☐ Healthy individual interested in future medical decisions

☐ Other: __________

(For medical staff only)
Please select your specialty or main field of activity:

☐ Anesthesiology

☐ Cardiology

☐ Pulmonology

☐ Internal Medicine

☐ Surgery

☐ Endocrinology

☐ Geriatrics

☐ Oncology

☐ Other: __________

Years of experience in the medical field:
[Free text]

Have you been involved in palliative care?

☐ Yes

☐ No

Have you participated in decisions regarding Artificial Nutrition and Hydration (ANH) at the end of life?

☐ Yes

☐ No

SECTION 3. ATTITUDES TOWARD ANH

To what extent do you agree with the following statements?
(Strongly disagree – Disagree – Neutral – Agree – Strongly agree)

- ANH extends life
- ANH improves quality of life at the end of life
- ANH prevents complications such as pressure ulcers or infections
- ANH should be provided regardless of the patient's clinical condition
- Continuing ANH for an unconscious patient is justified if requested by the family
- Withdrawing ANH for an unconscious patient is justified if requested by the family
- Withdrawing ANH is equivalent to euthanasia
- Initiating ANH without patient consent is justified in emergency situations
- Withdrawing ANH without patient consent is justified in emergency situations

SECTION 4. DECISION-MAKING FACTORS

How important are the following factors when deciding on ANH?
(Not important at all – Slightly important – Neutral – Important – Very important)

- Nutritional status
- Estimated life expectancy
- Patient's expressed wishes
- Family's opinion
- Quality of life
- Comorbidities
- Risk of pulmonary aspiration
- Type of nutrition (oral vs. enteral/parenteral)

SECTION 5. PERCEPTIONS OF ANH

To what extent do you agree with the following statements?
(Strongly disagree – Disagree – Neutral – Agree – Strongly agree)

- ANH may provide comfort
- ANH may cause suffering
- ANH may not provide meaningful benefit in terminal illness
- ANH should always be provided

SECTION 6. PERSONAL EXPERIENCE

Have you ever had a disagreement with the medical team regarding ANH at the end of life?

☐ Yes

☐ No

SECTION 7. PERSONAL PREFERENCES

Which of the following best reflects your wishes regarding feeding if you are no longer able to decide?

☐ I would like to receive food (solid or liquid) as long as possible

☐ I would prefer easily digestible or natural foods

☐ I would prefer my favorite foods adapted for feeding (e.g., blended)

☐ I would prefer sweet beverages (e.g., juices, honey)

☐ I would prefer hydration only (without nutrition)

☐ I would not want artificial feeding

☐ I would prefer that my family or doctors decide

☐ I am unsure / I have not thought about this

☐ Other: __________

SECTION 8. DECISION PROCESS

How should decisions regarding ANH be made?

☐ Individually (by the attending physician)

☐ Interdisciplinary (medical team + family)

☐ Based primarily on the patient's wishes

SECTION 9. EDUCATION

Do you feel you have sufficient ethical and legal training to participate in such decisions?

☐ Yes

☐ No

☐ Not sure

SECTION 10. CLINICAL GUIDELINES

What is your opinion regarding clinical guidelines on ANH?

☐ I agree with the guidelines and find them adequate

☐ The guidelines are clear and easy to understand

☐ The guidelines are useful but should be adapted to each case

☐ The guidelines lack specificity

☐ I disagree with stopping ANH regardless of guidelines

☐ I follow guidelines but adapt decisions to the clinical context

☐ I am not aware of relevant guidelines

☐ Decisions should be guided primarily by patients/families rather than guidelines

☐ I do not know enough to form an opinion

SECTION 11. LEGAL FRAMEWORK

What is your opinion regarding legislation on ANH?

☐ The legislation is clear and adequate

☐ The legislation is unclear

☐ The legislation is too restrictive

☐ I disagree with stopping ANH even if legally allowed

☐ There is a need for clearer legal regulation

☐ Decisions should be guided by patient/family wishes rather than law

☐ I am not familiar with the legislation

SECTION 12. TRAINING NEEDS

Do healthcare professionals need additional training in ANH decision-making and communication?

☐ Yes, it is essential

☐ Yes, especially for communication and informed consent

☐ Yes, but integrated into existing programs

☐ No, current training is sufficient

☐ I am unsure / I have no experience

SECTION 13. OPEN RESPONSE

Would you like to share a relevant personal experience regarding ANH at the end of life?

[Free text]
